# A review of guidance on fish consumption in pregnancy: is it fit for purpose?

**DOI:** 10.1017/S1368980018000599

**Published:** 2018-03-26

**Authors:** Caroline M Taylor, Pauline M Emmett, Alan M Emond, Jean Golding

**Affiliations:** Centre for Child and Adolescent Health, Population Health Sciences, Bristol Medical School, University of Bristol, Oakfield House, Oakfield Grove, Bristol BS8 2BN, UK

**Keywords:** Mercury, Fish, Pregnancy, Guidelines, Advisories, Review

## Abstract

**Objective:**

Public health messages to reduce Hg exposure for pregnant women have focused exclusively on advice on fish consumption to limit Hg exposure, with little account being taken of the positive contribution of fish to nutritional quality. The aim of the present review was to compare and contrast the content and presentation of national guidelines on fish consumption in pregnancy, and comment on their evidence base and impact on consumption.

**Design:**

We searched for national and international guidelines on fish consumption in pregnancy using Internet search strategies. The detailed content and style of presentation of the guidelines were compared. The evidence base for the guidelines, and evidence for the impact of the guidelines on fish consumption levels, were assessed.

**Results:**

We identified nineteen national guidelines and three international guidelines. There was great variation in the content, complexity and presentation style. The guidelines were based largely on the Hg content of fish with far less consideration being given to the positive beneficial effects of nutrients provided by fish. The complexity of the guidelines may lead to pregnant women reducing their fish intake, or not eating fish at all.

**Conclusions:**

Guidelines on fish consumption in pregnancy should take the beneficial effects of fish into account. Guidelines need to be clear and memorable, and appropriately disseminated, to achieve impact. Guidelines could include visual rather than narrative content. Use of technology, for example apps, could enable women to record their fish consumption in real time and log compliance with guidance over a week or other time period.

When women become pregnant, they are likely to receive a great deal of information on foods to avoid or limit. Inevitably, this advice will vary from country to country, but several countries provide detailed information specifically on types of fish to limit and those to avoid completely. This advice relates mainly to the Hg content of fish, with the aim of reducing the exposure of the pregnant woman and avoiding adverse effects on the neurodevelopment of the fetus.

Hg is a widespread environmental toxin. It is present in the environment through natural processes such as volcanic activity and the weathering of rocks, but also through anthropogenic activities such as mining, smelting, power generation and manufacturing^(^ ^1^ ^,^ ^2^ ^)^. Exposure can also occur through dental amalgams^(^ ^3^ ^)^, cosmetics^(^ ^4^ ^)^ and some food items^(^ ^5^ ^)^, primarily fish, in which it accumulates from contaminated aquatic environments^(^ ^6^ ^)^. Acute toxicity in man is associated with severe neurological symptoms and ultimately death^(^ ^7^ ^,^ ^8^ ^)^. However, there is not thought to be any lower limit for adverse effects. Since Hg passes through the placenta^(^ ^9^ ^,^ ^10^ ^)^, the fetus is assumed to be vulnerable to its toxic effects, which are amplified by the sensitivity of the rapidly developing nervous system. It is therefore recommended that pregnant women minimise exposure to Hg.

Because Hg levels in aquatic environments and local fish species vary, many countries have produced their own guidance. However, little is known about fish consumption in the adult female populations of many of these countries, and less still about the impact of the advice on consumption of fish during pregnancy. The aims of the present review were to: (i) identify, summarise and compare national and international guidelines on fish consumption during pregnancy, as well as for women planning pregnancy or breast-feeding; (ii) appraise the evidence base for the guidelines; (iii) comment on the data available on fish consumption in women of childbearing age and during pregnancy; and (iv) provide some ideas for research and other activities that would inform improvements in guidance and dissemination to ensure maximum benefit to the developing fetus.

## Current national and international guidelines

### Search strategy

To identify guidelines on fish consumption in pregnancy, three search strategies were employed: (i) keywords were used for Internet searches (‘Pregnancy’ OR ‘Pregnant’, ‘Mercury’ ‘Fish’, ‘Guideline’ OR ‘Advice’); (ii) the FAO’s website on dietary guidelines^(^ ^11^ ^)^ was explored country by country to check for relevant guidance on fish consumption; and (iii) the European Food Safety Authority’s (EFSA) report on the health benefits of seafood^(^ ^12^ ^)^ includes a list of countries that have dietary guidelines – these guidelines were accessed and searched for specific advice relating to pregnancy. This yielded international guidelines from three organisations (Table 1) and national guidelines from nineteen countries (two in North America, two in Australasia, twelve in Europe and three in Asia; Table 2). All were direct advice from national or government agencies, except the guidance from Japan, which was a translation available on the Internet, and the Korean guidelines, which were reported in a newspaper article. A web-based translation service was used where necessary. Several other countries provide guidance for adults but not for pregnant women in particular, such as Greece (5–6 portions/week)^(^ ^13^ ^)^, Brazil (include fish as part of a varied diet)^(^ ^14^ ^)^ and India (include fish as part of a varied diet)^(^ ^15^ ^)^.

**Table 1:** Recommendations from multinational organisations on fish consumption for pregnant women related to mercury

Organisation	Guideline
International Federation of Gynecology and Obstetrics (2015)^(^ ^18^ ^)^	1–2 meals of oily fish per week Avoid large predatory fish such as swordfish, marlin, tuna, shark, orange roughy, king mackerel, bigeye or ahui tuna and tilefish; cold smoked seafood; undercooked or raw fish Limit intake of bass, carp, Alaskan cod, halibut, mahi mahi, freshwater perch, monkfish, sea trout and snapper to 1–2 meals per week
European Food Safety Authority	
2014^(^ ^12^ ^)^	About 1–2 servings per week up to 3–4 servings per week in pregnancy associated with better neurodevelopmental outcomes in offspring compared with no consumption. Observed benefits may depend on maternal status of nutrients with an established role in the development of the central nervous system of the fetus (e.g. DHA, iodine)
2015^(^ ^16^ ^)^	Women of childbearing age should gain benefits of eating fish by increasing the consumption of species lower in Hg but not exceed the tolerable weekly intake of 1·3 µg/kg body weight per week Not possible to make general recommendations on fish consumption across Europe because of differences in species of fish consumed in different countries
FAO/WHO (2011)^(^ ^17^ ^)^	‘Among women of childbearing age, pregnant women and nursing mothers, considering benefits of DHA versus risks of methylmercury, fish consumption lowers the risk of suboptimal neurodevelopment in their offspring compared with not eating fish in most circumstances evaluated’ Large regional variations in Hg content of fish acknowledged, with call for specific information on levels of contamination

**Table 2:** Recommendations on fish consumption related to mercury in pregnancy in individual countries

Country	Year	Do not eat	Limit	Eat freely/General advice
North America				
USA^(^ ^25^ ^)^ *,†	2017	For women of childbearing age (16–49 years) and especially during pregnancy and breast-feeding: ∙ King mackerel, marlin, orange roughy, shark, swordfish, tilefish (Gulf of Mexico), tuna (bigeye) ∙ Additional warning on large carp, catfish, trout and perch caught by family and friends: check with state advisories (if no advisory in place, eat only 1 serving and no other fish that week)	For women of childbearing age (16–49 years) and especially during pregnancy and breast-feeding: Eat 2–3 servings per week†† from ‘Best choices’ or 1 serving per week†† from ‘Good choices’ list ∙ ‘Good choices’. Eat 1 serving/week: bluefish, buffalo fish, carp, Chilean sea bass/Patagonian toothfish, grouper, halibut, mahi mahi/dolphinfish, monkfish, rockfish sable fish, sheepshead, snapper, Spanish mackerel, striped bass (ocean), tilefish (Atlantic Ocean), tuna (albacore/white tuna, canned and fresh/frozen), tuna (yellowfin), white croaker/pacific croaker ∙ ‘Best choices’. Eat 2–3 servings/week: anchovy, Atlantic croaker, Atlantic mackerel, black sea bass, butterfish, catfish, clam, cod, crab, crawfish, flounder, haddock, hake, herring, lobster (American and spiny), mullet, oyster, Pacific chub mackerel, perch (freshwater and ocean), pickerel, plaice, pollock, salmon, sardine, scallop, shad, shrimp, skate, smelt, sole, squid, tilapia, trout (freshwater), tuna (canned light – includes skipjack), whitefish, whiting)	For women of childbearing age (16–49 years) and especially during pregnancy and breast-feeding: ∙ Eat a variety of fish
Canada^(^ ^32^ ^)^ †	ND	–	When trying to get pregnant/during pregnancy/during breast-feeding: ∙ Limit some predatory fish to less than 150 g per month: tuna (fresh and frozen), shark, swordfish, marlin, orange roughy, escolar ∙ Limit canned (white) albacore tuna to no more than 300 g per week	When trying to get pregnant/during pregnancy/during breast-feeding: ∙ Eat at least 150 g of cooked fish per week ∙ Vary type of fish eaten ∙ No limit on other types of canned tuna (e.g. skipjack, yellowfin, tongol)
Australasia				
Australia/New Zealand^(^ ^29^ ^)^	2011	–	When trying to get pregnant/during pregnancy: ∙ 2–3 servings per week of any fish and seafood not listed below; or ∙ 1 serving (150 g cooked) per week of orange roughy (sea perch) or catfish and no other fish that week; or ∙ 1 serving (150 g cooked) per fortnight of shark (flake), marlin or broadbill/swordfish, and no other fish that fortnight	–
New Zealand^(^ ^68^ ^)^	ND	–	During pregnancy: ∙ No more than 3 servings (150 g/serving) per week: uncanned wild-caught (not farmed) salmon, uncanned albacore tuna or mackerel, kahawai, red cod, orange roughy and ling ∙ Once every 2 weeks (or not at all if eating other types of fish): school shark, southern bluefin tuna, marlin and trout from geothermal regions and Lake Rotomahana ∙ Bluff and pacific oysters, queen scallops**	During pregnancy: ∙ Canned tuna (skipjack or albacore), canned salmon, mackerel, sardines, farmed salmon, terakihi, blue cod, hoki, john dory, monkfish, warehou, whitebait, flat fish (e.g. flounder)
Europe				
UK^(^ ^20^ ^–^ ^23^ ^)^ ‡	2017, 2015, 2015, 2015	When trying to get pregnant/during pregnancy: ∙ Shark, swordfish, marlin ∙ Raw shellfish¶	When trying to get pregnant: ∙ Limit amount of tuna‡‡ to not more than (i) two tuna steaks per week (each about 140 g cooked weight or 170 g when raw) or (ii) four medium-sized cans of tuna per week (about 140 g per can when drained) During pregnancy: ∙ Limit amount of tuna‡‡ to not more than (i) two tuna steaks per week (each about 140 g cooked weight or 170 g when raw) or (ii) four medium-sized cans of tuna per week (about 140 g when drained) ∙ Limit amount of oily fish‡‡ to not more than two portions per week (oily fish includes fresh tuna, salmon, trout, mackerel, herring, sardines, pilchards) ∙ Limit amount of other fish to not more than two portions per week (other fish includes dogfish (rock salmon), sea bass, sea bream, turbot, halibut, crab)§§ During breast-feeding: ∙ Not more than two portions of oily fish per week ∙ No limit on canned tuna ∙ Not more than one portion per week of shark, swordfish or marlin§§	Eat at least two portions of fish per week (at least one should be oily fish but no more than two) No need to limit or avoid other types of white and non-oily fish such as cod, haddock, plaice, coley, skate, hake, flounder, gurnard††
Germany^(^ ^69^ ^)^	2013	During pregnancy: ∙ Carnivorous fish such as tuna and swordfish ∙ Smoked fish	–	During pregnancy: ∙ Two portions of fish per week, with one portion of oily fish (mackerel, herring, sardines or salmon) ∙ For women who do not eat seafood regularly, it is recommended that they take a supplement containing DHA
France^(^ ^30^ ^)^	2016	During pregnancy/during breast-feeding: ∙ Shark, lamprey, swordfish, marlin, siki	During pregnancy/during breast-feeding: ∙ Limit to 150 g per week: monkfish or angler fish, Atlantic wolf-fish, bonito, eels and elvers, emperor, orange roughy, rosy soldierfish, grenadier, Atlantic halibut, megrim, mullet, pike, plain bonito, poor cod, Portuguese dogfish, rays (skate), redfish, Atlantic sailfish, silver and black scabbardfish, sea bream, pandora, black or striped escolar, oilfish, snake mackerel, sturgeon, tuna, etc.	Eat fish twice per week, including oily fish (salmon, mackerel, sardines, anchovies, smoked trout, herring, etc.) Eat a variety of fish
Spain^(^ ^70^ ^)^	ND	During pregnancy: ∙ Swordfish, fresh tuna, pike, shark ∙ Raw fish, smoked fish, oysters, clams, raw mussels	–	Eat a wide variety of fish Eat fish 3–4 times per week (mainly oily fish)
Italy^(^ ^71^ ^)^	2016	–	During pregnancy: ∙ 1–2 up to 3–4 servings of fish per week; prefer small fish such as sardines, mackerel and anchovies with high *n*-3 fatty acid content During breast-feeding: ∙ 2 servings of fish per week	–
The Netherlands^(^ ^19^ ^)^	2015	During pregnancy: ∙ Predatory fish such as sharks, king mackerel, swordfish, tilefish, tuna (except canned tuna) ∙ Wild eels and mitten crabs from Dutch waters	–	Eat fish twice per week, including at least one portion of oily fish
Ireland^(^ ^31^ ^)^	2004	Shark, swordfish, marlin	During pregnancy: ∙ Not more than two portions of oily fish per week ∙ Not more than two fresh tuna steaks per week or four cans of tuna per week	Eat two portions per week, including one portion of oily fish
Sweden^(^ ^24^ ^)^	2008	–	During pregnancy: ∙ Eat maximum 2–3 times per year: Atlantic halibut, burbot, perch, pike, pikeperch, ray, shark, swordfish, tuna (fresh/frozen) ∙ Eat maximum 2–3 times per year**: Baltic herring, fermented herring, salmon and salmon trout from the Baltic Sea, Lakes Vaneren and Vattern, and char from Lake Vattern	Eat fish 2–3 times per week Eat a variety of fish Safe to eat: all farmed fish, Alaska pollock, anchovies, blue mussels, canned tuna, catfish, cod, crab (white flesh), crayfish, fishballs, fish-fingers, flounders/dabs, haddock, hake, herring (including pickled), hoki, lobster, mackerel, plaice, prawns, saithe, salmon, trout, sardines, scallops, stockfish, tilapia, whitefish, etc.
Finland^(^ ^34^ ^)^	ND	During pregnancy/while breast-feeding: ∙ Pike ∙ Raw-cured or smoke-cured fish, raw fish	During pregnancy: ∙ Fish from the Baltic Sea, such as salmon, trout and large Baltic herrings (>17 cm), should not be eaten more than once or twice per month	During pregnancy: ∙ Eat a variety of fish (such as saithe, trout, rainbow trout, Arctic char, whitefish and vendace) two to three times per week
Norway^(^ ^33^ ^)^	2011	Shrimp, Greenland halibut >3 kg, freshwater fish (pike, perch >25 cm, trout >1 kg, char >1 kg), exotic fish (hai, swordfish, skater, fresh tuna), fish liver and fish liver products Avoid brown crab meat, digestive tract in scallops, kidneys of horse mussels** Additional advisories online regarding on fish caught by friends and family: preferably avoid	–	Eat fish 2–3 times per week (300–450 g) (at least 200 g should be oily fish: salmon, trout, mackerel, herring) Canned tuna
Denmark^(^ ^72^ ^)^	ND	During pregnancy: ∙ Canned white tuna or albacore When trying to get pregnant/during pregnancy/during breast-feeding: ∙ Large predatory fish such as tuna, rockfish, halibut, escolar, swordfish, herring, shark, perch, pike and pikeperch	When trying to get pregnant/during pregnancy/during breast-feeding: ∙ Not more than one serving (125 g) of salmon from the Baltic Sea per month**	During pregnancy: ∙ Eat 350 g per week, 200 g of which should be oily and from a variety of fish (plaice, red tuna, flounder, cod, haddock, hake, squid, fish eggs, and oily fish such as mackerel, herring and farmed salmon)
Iceland^(^ ^73^ ^)^	ND	Pregnancy: ∙ Raw fish, cured fish, cold-smoked fish, dried fish, sushi, pickled whale, cod liver, shark, swordfish, large halibut, fulmar, fulmar eggs	During pregnancy: ∙ Eat no more than one serving per week of tuna fish steak, orange roughy ∙ Eat no more than two servings per week of canned tuna, guillemot eggs, minke whale meat	Eat fish twice per week
Asia				
Israel^(^ ^74^ ^)^	2017	During pregnancy: ∙ Large fish such as shark, swordfish, king mackerel, tilefish, tuna steaks and white tuna (albacore) ∙ Raw and cold cured fish	–	Consume fish from locally available fish, including pond fish and canned light tuna. Eat a variety of fish
Japan^(^ ^27^ ^)^ §	2005	–	During pregnancy: ∙ Up to 80 g (average 1 meal) per 2 months: bottlenose dolphin ∙ Up to 80 g (1 meal) per 2 weeks: short-finned pilot whale ∙ Up to 80 g (1 meal) per week: swordfish, bluefin tuna, bigeye tuna, finely striate buccinum║║, Baird’s beaked whale, sperm whale ∙ Up to 160 g (average 2 meals) per week: yellowback sea bream, marlin, Hilgendorf’s saucord, southern bluefin tuna, blue shark, Dall’s porpoise	Tuna species other than those listed plus canned tuna
Korea^(^ ^75^ ^)^ ║	2013	Tuna, raw fish	–	During pregnancy: ∙ Eat fish daily

ND, not dated.

* Includes an infographic illustrating the categories and portion sizes. Endorsed by the American College of Obstetricians and Gynaecologists with the additional advice that pregnant women should avoid raw and undercooked seafood^(^ ^76^ ^)^.

† Other guidelines and advisories for North American populations are shown in Oken *et al*. (2012)^(^ ^28^ ^)^.

‡ Based on the NHS Choices website ‘Should pregnant and breastfeeding women avoid some types of fish?’^(^ ^21^ ^)^.

§ Online translation from original.

║ Newspaper article reporting advice from the Korea Health Promotion Foundation.

¶ Raw shellfish is not advised in pregnancy as it can be a microbiological hazard, but cooked shellfish can be eaten freely^(^ ^22^ ^)^.

** High levels of Cd, Pb, polychlorinated biphenyls and/or dioxins.

†† A serving is defined as 4 oz for an adult (about 110 g). The guidance also applies to ‘young children’, who are advised to eat 1–2 servings of fish/week starting at age 2 years (child’s serving defined as 2 oz (about 55 g)).

‡‡ Under the guidance, canned tuna does not count as oily fish so is not included in the maximum of two portions of oily fish per week. However, because of the higher Hg level in tuna, if eating canned tuna, the advice is not to pick fresh tuna as one of the tally of oily fish.

§§ The NHS Choices website ‘Your pregnancy and baby guide. Foods to avoid in pregnancy’ omits these points^(^ ^20^ ^)^.

║║ A type of whelk.

### Comparison of guidance

The three international guidelines identified (Table 1) were from EFSA^(^ ^16^ ^)^ (European countries), FAO/WHO^(^ ^17^ ^)^ (worldwide) and the International Federation of Gynecology and Obstetrics (worldwide)^(^ ^18^ ^)^. The first two are general in nature, and indeed EFSA notes that it is ‘not possible to make general recommendations on fish consumption across Europe because of differences in species of fish consumed in different countries’. The FAO/WHO, however, takes a rather different viewpoint from any of the other guidelines in moving the emphasis away from adverse effects of fish consumption, stating that the benefits of DHA from fish consumption outweigh the adverse effects of methylmercury and that consumption of fish lowers the risk of suboptimal neurodevelopment in the offspring.

The nineteen national guidelines (Table 2), which are generally from developed countries, vary from relatively simple and memorable (e.g. the Netherlands^(^ ^19^ ^)^) to highly complex (e.g. UK^(^ ^20^ ^–^ ^23^ ^)^). Some refer to pregnancy only while others extend their recommendations to include women who are planning to become pregnant and/or those who are breast-feeding. Some reflect local aquatic conditions, fish species and fish consumption habits (e.g. Sweden advises against particular fish species from the Baltic Sea^(^ ^24^ ^)^; the USA advises checking state advisories on specific larger fish caught by friends and family^(^ ^25^ ^,^ ^26^ ^)^). The Japanese guidelines are markedly different from those of the other countries in that they provide advice mainly on consumption of dolphin and whale species rather than fish^(^ ^27^ ^)^. Most national guidelines give categorised advice, sometimes in great detail, about fish to avoid, limit or eat freely. In contrast, the USA provides a list of ‘Choices to avoid’, together with ‘Best choices’ (2–3 servings/week) and ‘Good choices’ (1 serving/week)^(^ ^25^ ^)^. The UK advice relating to three physiological states (planning pregnancy, pregnant, breast-feeding) is particularly specific for each condition^(^ ^20^ ^–^ ^23^ ^)^. Twelve additional sources of guidance and advisories on fish consumption related to contaminant exposure for North American populations in addition to that of the US Environmental Protection Agency were identified in 2012 by Oken *et al*.^(^ ^28^ ^)^. Some of the guidance refers to hazards other than Hg (e.g. raw fish can contain parasitic anisakid nematode, which is not killed by cold curing but can be killed by freezing or cooking; raw shellfish can be contaminated with bacteria or viruses that can cause food poisoning; some fish species and shellfish can be contaminated with Cd, as well as polychlorinated biphenyls and dioxins).

The guidelines are relatively consistent in the species of fish that pregnant women are advised not to eat: these tend to be the predatory species prevalent and consumed in each county. Both the USA and UK, for example, include marlin, shark and swordfish in the ‘do not eat’ list, but the USA also includes some additional species (king mackerel, tilefish, etc.). The advice relating to tuna, however, is particularly diverse, with some guidelines distinguishing between different types of tuna (e.g. the USA requires distinction between albacore/white tuna and yellowfin tuna, which are classified as ‘Good choices’, and canned light tuna including skipjack, which is classified as ‘Best choices’^(^ ^25^ ^)^; the UK, on the other hand, distinguishes between fresh and canned tuna, each of which has an advised maximum limit per week during pregnancy and when trying to get pregnant; during breast-feeding, however, canned tuna is unlimited but there is no specific advice on fresh tuna^(^ ^20^ ^–^ ^23^ ^)^).

In nearly all cases, careful compliance with the guidelines would require women to keep a tally of consumption of particular species over the course of a week (e.g. the USA^(^ ^25^ ^)^, Australia/New Zealand^(^ ^29^ ^)^, UK^(^ ^20^ ^–^ ^23^ ^)^, France^(^ ^30^ ^)^, Ireland^(^ ^31^ ^)^), two weeks (Australia/New Zealand^(^ ^29^ ^)^), four weeks (Canada^(^ ^32^ ^)^) or even two months (Japan^(^ ^27^ ^)^). They also require that the woman is confident in remembering or accessing a list of different species of fish and being able to identify different species of fish (e.g. France names nearly thirty species in the ‘limited’ category^(^ ^30^ ^)^ and the USA includes nearly seventy species in its lists of ‘Good choices’ and ‘Bad choices’^(^ ^25^ ^)^). The Australian/New Zealand guidelines suggest asking the retailer or restaurant about the type of fish on offer if in doubt^(^ ^29^ ^)^. Strict adherence to some guidelines would also require a pocket tape measure and/or weighing scales^(^ ^33^ ^,^ ^34^ ^)^.

The presentation and content of current advice for the USA is rather different from that of other countries (Table 2), which usually include ‘traditional’ headings of fish to limit, fish to avoid and fish to eat freely. The US guidance appears on a Food and Drug Administration/Environmental Protection Agency webpage in an infographic format featuring blocks of information for each of the types of choice (‘Choices to avoid’/‘Good choices’/‘Bad choices’). It also features pictorial guidance on the size of a portion of fish for an adult and for a child based on hand size. Advice to refer to state advisories for locally caught fish is not signposted specifically from the infographic. The infographic is followed by a ‘questions and answers’ section providing detailed information on using the chart, portion sizes, specific information for children, nutrients and contaminants in fish, and more detailed information on tuna; this section includes further information on fish caught by friends and family with a link to the Environmental Protection Agency website on state advisories^(^ ^35^ ^)^. Despite the differences in presentation style, it shares complexity with other guidance in requiring memory and the fish-identification skills referred to earlier.

## Evidence for beneficial effects of fish on child health and development

The guidance in the UK was developed from the recommendations of the Scientific Advisory Committee on Nutrition published in 2004. These were based on calculation of the Hg content of fish that would result in exposure at a provisional tolerable weekly intake of 1·6 µg/kg body weight for pregnant women as being sufficient to protect against adverse effects on neurodevelopment in the fetus (3·3 µg/kg body weight for breast-feeding women)^(^ ^36^ ^)^. Similar approaches have been adopted by other countries, but they fail to take into account the potential risk-payoff from fish consumption^(^ ^37^ ^–^ ^39^ ^)^: fish is a rich source of protein, as well as other nutrients required for fetal neurodevelopment including iodine, Se, choline, vitamin D and long-chain *n*-3 fatty acids. Iodine levels particularly have been shown to be low in pregnant women in the UK^(^ ^40^ ^)^ and this has been associated with adverse effects on offspring IQ (intelligence quotient)^(^ ^41^ ^)^. Indeed, studies from a UK birth cohort have shown evidence of a positively beneficial effect of eating fish in pregnancy on a range of developmental outcomes in the child^(^ ^42^ ^–^ ^44^ ^)^. Evidence for risk is also sometimes based on studies from the Faroe Islands, where Hg exposure is derived from consumption of pilot whales rather than fish^(^ ^45^ ^)^. The evidence for beneficial effects of fish consumption on many aspects of maternal health and child development has increased in recent years. In the UK, the Avon Longitudinal Study of Parents and Children (ALSPAC; observational birth cohort) includes data on prenatal measures of Hg exposure, together with maternal fish consumption and a range of childhood outcome indicators. In this cohort, consumption of two to three portions of fish per week is associated with beneficial effects on child development, suggesting that limiting fish intake might actually be detrimental^(^ ^37^ ^)^. Fish consumption made only a small contribution to the variation in blood levels of Hg during pregnancy^(^ ^46^ ^)^. There was no effect on the likelihood of the baby being born with a low birth weight or preterm; indeed, birth weight was lower in the babies of mothers who did not eat fish during pregnancy, suggesting that fish consumption has a beneficial effect on birth weight^(^ ^47^ ^)^. Other measures of child development, such as child behaviour, social, motor and communication skills, and IQ, similarly showed no association with prenatal Hg exposure^(^ ^42^ ^–^ ^48^ ^)^. These findings have been substantiated by similar evidence from outside the UK, for example from the Seychelles Development Study, where there is daily fish consumption and Hg exposure levels are about ten times higher than typical exposures in the USA: a variety of neurodevelopmental tests have been applied at ten age points in 24 years of follow-up without any evidence of associations with prenatal exposure to Hg^(^ ^49^ ^–^ ^51^ ^)^. Similarly, no associations of prenatal fish intake or Hg exposure with cognitive outcomes were found in children aged about 7 years in Project Viva in the USA, despite adjustment for long-chain *n*-3 fatty acids (DPA+EPA) and Se^(^ ^52^ ^)^. In the Norwegian Mother and Child Cohort Study, seafood intake was positively associated with birth weight, whereas Hg exposure was negatively associated, suggesting that the balance of the risks and benefits of seafood might need further quantification^(^ ^53^ ^)^. However, unlike the other studies described, prenatal Hg exposure was calculated from dietary intakes (FFQ) and may not represent Hg exposure in the same way. Other studies have shown similar positive associations of child neurodevelopment with prenatal fish intake^(^ ^54^ ^)^.

## How much fish do pregnant women eat?

It would therefore seem to be disadvantageous if the guidelines had the unintended consequence of reducing fish consumption in pregnant women. To understand the impact of these guidelines on fish consumption, it is necessary first to have accurate nationally representative data specifically from pregnant women. Fish consumption in women of childbearing age and pregnant women has consistently been shown to be below recommended levels. In a compilation of data on pregnant women from nineteen European birth cohort studies with recruitment from 1996 to the date of publication (2014)^(^ ^55^ ^)^, the median fish intake ranged from 0·4 times/week in the Netherlands (the Generation R study) to 4·5 times/week in Spain (Childhood and Environment Project (INMA)). The median oily fish intakes in Italy (Genetic and Environment: Prospective Study on Infancy in Italy (GASPII)), Portugal (Generation XXI), Spain (INMA) and Poland (Polish Mother and Child Cohort Study (REPRO-PL)) were more than twice the overall median intake of 0·5 times/week. Portion sizes of different fish types varied from 100 to 150 g across cohorts that included this information. Thus, in fourteen of the nineteen studies, the median intake was less than 2–3 times/week, and no study reported an intake of oily fish of more than 1 time/week (six studies had no data on oily fish intake). A more recent (2017) compilation of seventeen cohorts in eleven European countries plus one cohort in the USA, which included some of the same studies as the previous compilation, found an overall consumption of 1·5 times/week (oily fish 0·6 times/week): women in all but three of the cohorts (Spain, INMA; Portugal, Generation XXI; Italy, NINFEA) ate fish less than 2 times/week, and none of the cohorts reported oily fish intake more than 1 time/week^(^ ^56^ ^)^.

More specifically, in the UK (where the recommended intake is for at least 2 portions fish/week with at least 1 portion oily fish/week; Table 2), women aged 19–64 years participating in the National Diet and Nutrition Survey (NDNS) from 2008 to 2012 ate a mean of 22 g fish/d (about 1 portion/week) including just 8 g oily fish/d (about 0·3 portion/week)^(^ ^57^ ^)^; mean consumption in pregnant women enrolled in ALSPAC was 235 g/week (about 1·5 portions/week) but 12 % ate no fish at all^(^ ^37^ ^)^; in the Southampton Women’s Study, total fish consumption was 1·8 times/week and oily fish consumption was about 0·5 times/week^(^ ^55^ ^,^ ^58^ ^)^. Findings in the USA are similar: women of childbearing age in the National Health and Nutrition Examination Surveys (NHANES) ate a median of 81 g/week (about 0·5 portion/week) and 23 % reported not eating any fish^(^ ^59^ ^,^ ^60^ ^)^; mean fish intake in pregnant women was 1·5 portions/week and 14 % never ate any fish. In Australia, mean intakes are a little higher at about 28 g/d^(^ ^61^ ^)^, but still fall far short of national recommended intakes (Table 2).

The methodology used in the surveys conducted is critical to the interpretation of studies on fish consumption: dietary recalls or dietary records are not ideal to capture an item that might be infrequently consumed and have the potential to underestimate fish/seafood intake. For example, in data compiled by EFSA, all country-level surveys were conducted with 24 h dietary recalls or dietary records and this was noted as being likely to ‘have the potential for overestimating the high ends of the distribution of fish/seafood consumption’^(^ ^16^ ^)^. EFSA also noted that conversion of values from daily to weekly to enable comparisons can magnify inaccuracies, as well as there being considerable between-country variation in mean portion size. Methods based on FFQ are likely to be more accurate for this type of low frequency food, but still present difficulties over the length and depth of detail in the questionnaire^(^ ^62^ ^)^.

## What evidence is there for the effect of guidelines?

Despite evidence of fish consumption below recommended levels in pregnancy, there has been very little research on the impact of the guidelines on consumption levels or consideration of how consumption levels could be optimised. Most public messages usually struggle to have impact, but there is some evidence that messages that are more ‘alarming’ achieve greater change. As an illustration of this effect, the US Food and Drug Administration issued an advisory notice on avoidance of predatory fish and limitation of consumption of all other fish in 2001 (before this time there was no specific guidance for pregnant women as Hg from commercial fish was not thought to pose any significant health threats). The result of the advisory, which was widely promoted, was a reduction in total fish consumption in pregnancy by about 0·4 portions/week during the year following the notice, with diminished consumption of dark-meat fish, canned tuna and white-meat fish^(^ ^63^ ^)^. Shimshack and Ward^(^ ^38^ ^)^ provided estimates of the observed effects of this advisory on Hg and *n*-3 fatty acid intakes and found that Hg intakes across the US population did fall by 17 %, but *n*-3 intakes also fell by 21 %, providing evidence of an unintended consequence. They attributed the fall in *n*-3 intake to ‘coarse information and broad behavioural guidance’ with lack of a detailed explanation of the recommendation that consumers select ‘a variety of other kinds of fish’^(^ ^38^ ^)^. The 2001 advisory was replaced in 2004 and again in 2017 (Table 3). For the 2004 guidance^(^ ^64^ ^)^, there is some evidence that in the face of confusing and complex guidelines, and the lack of readily available advice, many women gave up eating fish: analysis of focus groups for twenty-two pregnant women who ate <2 portions fish/week in the USA in 2009/10 showed that many of them had received advice to limit their fish intake and knew that fish could contain Hg. Because of this advice and a lack of knowledge about which types of fish were safer to eat, many of the women reported that they would rather avoid fish altogether than risk harm to themselves or their baby. They felt that advice from a doctor on eating fish and readily available information on which fish are safe to eat would have encouraged them to eat more fish^(^ ^65^ ^)^. There is direct evidence of lower intakes in pregnant women compared with non-pregnant women in Australia, where mean intakes were 28 g/d in pregnant women but significantly greater at 33 g/d in women who were not pregnant, trying to conceive or <1 year postpartum^(^ ^61^ ^)^.

**Table 3:** Changes in the US Food and Drug Administration (FDA)/Environmental Protection Agency guidelines on fish consumption for women planning to become pregnant and those who are pregnant or breast-feeding

Pre-2001	Hg from commercial fish consumption not considered to pose significant health threats and the benefits of seafood consumption outweigh the risks		
Advisory published in 2001*	Avoid large predatory fish. Limit consumption of all fish, including canned fish, to <12 oz/week‡. Eat a variety of other fish – including shellfish, canned fish, smaller ocean fish and farm-raised fish		
	Do not eat	Limit	General
Guidance published in 2004^(^ ^64^ ^)^	∙ Tilefish from the Gulf of Mexico ∙ Shark ∙ Swordfish ∙ King mackerel	White (albacore) tuna to 6 oz/week as part of 2 servings of fish per week In addition, limit fish caught from streams, rivers and lakes to 6 oz/week in the absence of specific advice from fish advisories on those waterbodies, but don’t eat any other fish that week	Eat up to 12 oz of a variety of fish and shellfish that are lower in Hg per week (2 servings) Choose fish lower in Hg: ∙ Salmon ∙ Shrimp ∙ Pollock ∙ Tuna (light canned) ∙ Catfish
	Do not eat	Limit	General
Draft advice released in 2014†	∙ Tilefish from the Gulf of Mexico ∙ Shark ∙ Swordfish ∙ King mackerel	White (albacore) tuna to 6 oz/week In addition, limit fish caught from streams, rivers and lakes to 6 oz/week in the absence of specific advice from fish advisories on those waterbodies^(^ ^26^ ^)^	Eat 8–12 oz of a variety of fish per week (2–3 servings) Choose fish lower in Hg: ∙ Salmon ∙ Shrimp ∙ Pollock ∙ Tuna (light canned) ∙ Tilapia ∙ Catfish ∙ Cod
	Do not eat	Good choices	Best choices
Guidance published in 2017^(^ ^25^ ^)^	∙ Tilefish from the Gulf of Mexico	Childbearing age (16–49 years) and especially during pregnancy and breast-feeding: Eat 2–3 servings per week from ‘Best choices’ or 1 serving per week from ‘Good choices’ list	
	∙ Shark ∙ Swordfish ∙ King mackerel ∙ Marlin ∙ Orange roughy ∙ Bigeye tuna	‘Good choices’: Bluefish, buffalo fish, carp, Chilean sea bass/Patagonian toothfish, grouper, halibut, mahi mahi/dolphinfish, monkfish, rockfish sable fish, sheepshead, snapper, Spanish mackerel, striped bass (ocean), tilefish (Atlantic Ocean), tuna (albacore/white tuna, canned and fresh/frozen), tuna (yellowfin), white croaker/pacific croaker	‘Best choices’: Anchovy, Atlantic croaker, Atlantic mackerel, black sea bass, butterfish, catfish, clam, cod, crab, crawfish, flounder, haddock, hake, herring, lobster (American and spiny), mullet, oyster, Pacific chub mackerel, perch (freshwater and ocean), pickerel, plaice, pollock, salmon, sardine, scallop, shad, shrimp, skate, smelt, sole, squid, tilapia, trout (freshwater), tuna (canned light – includes skipjack), whitefish, whiting)

In 2015 the American College of Obstetricians and Gynecologists issued guidance reflecting the 2014 FDA draft advice^(^ ^76^ ^)^; this was superseded by a practice advisory in 2017 in line with the 2017 FDA advice^(^ ^77^ ^)^.

* Cited in Shimshack and Ward^(^ ^38^ ^)^.

† First accessed 27 September 2015. Not available online when access attempted again on 5 October 2017 (originally available at http://www.fda.gov/Food/FoodborneIllnessContaminants/Metals/ucm393070.htm).

‡ About 330 g.

There is some evidence, however, that targeted education during pregnancy can result in increases in fish consumption. Fifty-five pregnant women in the USA who were low fish eaters (≤2 servings/month) were randomised to receive control messages, advice to eat low-Hg fish or advice to eat low-Hg fish plus coupons to buy fish: fish consumption increased in both intervention groups without an increase in blood Hg levels compared with baseline values^(^ ^66^ ^)^. Although this was a pilot study with small numbers of women, it does indicate that women are receptive and willing to increase their fish intake and can achieve this without increasing Hg exposure when given appropriate targeted advice.

## Summary and conclusion

There is great variation in the content, complexity and presentation style of guidance for pregnant women on fish consumption between countries. This partly reflects local environmental conditions, species availability and consumption preferences, and to some extent local preferences for delivery of public health messages. The guidelines have largely been based on the Hg content of fish with far less consideration being given to the positive beneficial effects of nutrients provided by fish. There is evidence for low levels of several nutrients provided by fish – notably iodine – in pregnant women and it is essential that pregnant women are given balanced advice to make informed choices.

There is some evidence that pregnant women find the advice confusing and prefer to give up eating fish altogether rather than take the risk of harm. There is general agreement that pregnant women should eat at least two portions of fish per week, but this message is not always clear and prominent. Fish consumption falls below this level in many countries and this may have adverse effects on offspring health and development. Guidance needs to be clear, simple and memorable, and appropriately disseminated, to achieve impact^(^ ^67^ ^)^. Guidance could include visual rather than narrative content. Use of technology, for example the development of apps, could enable women to record their fish consumption in real time and give feedback on compliance with guidance over a week or other time period.
